# Impact of hydrothermal and mechanical processing on dissolution kinetics and rheology of oat β-glucan

**DOI:** 10.1016/j.carbpol.2017.02.077

**Published:** 2017-06-15

**Authors:** Myriam M.-L. Grundy, Janina Quint, Anne Rieder, Simon Ballance, Cécile A. Dreiss, Peter J. Butterworth, Peter R. Ellis

**Affiliations:** a Biopolymers Group, Diabetes and Nutritional Sciences Division, King’s College London, Franklin-Wilkins Building, 150 Stamford Street, London SE1 9NH, UK; b Institute of Food Research, Norwich Research Park, Colney, Norwich NR4 7UA, UK; c Department of Nutritional Sciences, University of Vienna, Althanstraße 14 (UZA II), 1090 Vienna, Austria; d Nofima, Norwegian Institute for Food, Fisheries and Aquaculture Research, PB 210, N-1431 Ås, Norway; e Institute of Pharmaceutical Science, King’s College London, Franklin-Wilkins Building, 150 Stamford Street, London SE1 9NH, UK

**Keywords:** Oat β-glucan, Solubility, Oat structure, Viscosity flow behaviour, Molecular weight

## Abstract

Oat mixed-linkage β-glucan has been shown to lower fasting blood cholesterol concentrations due notably to an increase in digesta viscosity in the proximal gut. To exert its action, the polysaccharide has to be released from the food matrix and hydrated. The dissolution kinetics of β-glucan from three oat materials, varying in their structure, composition and degree of processing, was investigated by incubating the oats at 37 °C over multiple time points (up to 72 h). The samples were analysed for β-glucan content, weight-average molecular weight and rheological behaviour. Regardless of the materials studied and the processing applied, the solubilisation of β-glucan was not complete. Mechanical and hydrothermal processing led to differences in the viscosity flow curves of the recovered solutions, with the presence of particulates having a marked effect. This study revealed that the structure and processing methods applied to oat materials resulted in varied and complex rheological properties, especially when particulates are present.

## Highlights

• β-Glucan release from oat cell walls during incubation was not complete.
• Processing of oats affects the rate and extent of β-glucan release and solution rheology.
• The rheology of β-glucan solutions varied depending on oat composition and presence of particulates.
• Variations in β-glucan solubility have important implications for physiological activity.

## 1 Introduction

The common oat grain (*Avena sativa* L.) is consumed by humans mainly as breakfast cereals, comprising whole grain flour or flakes, which can be eaten either as porridge after heating in water/milk or in the form of ready-to-eat cereals, such as muesli and granola (Webster, 2011). Oat flour is often used as an ingredient in bread, muffins, granola bars, biscuits and snack bars. Typical commercial products vary in the size and shape of the oat particles they contain. An important component of oats is β-glucan, which is composed of a mixed-linkage linear polymer of (1 → 3)(1 → 4)-β-d-glucan. This polymer is a water-soluble dietary fibre that is considered to have nutritional benefits, such as lowering plasma cholesterol concentrations.

β-Glucan is located in the endosperm cell walls of oats, with particularly rich concentrations found in the sub-aleurone layers, i.e., the endosperm tissue located adjacent to the aleurone layer (Miller & Fulcher, 2011). Commercial oat bran can contain significant concentrations of β-glucan because the milled bran comprises large amounts of adhering endosperm, including sub-aleurone tissue. The β-glucan content of oat varies depending on genotype and environmental conditions during growth and ranges from ∼2.2 to 7.8% (Lazaridou, Biliaderis, & Izydorczyk, 2007). It is a polydisperse polysaccharide with reported values of average molecular weight (MW) between ∼0.1 and 2.5 million g/mol (Åman, Rimsten, & Andersson, 2004; Andersson & Börjesdotter, 2011; Beer, Wood, & Weisz, 1997; Doublier & Wood, 1995; Johansson, Virkki, Maunu, Lehto, Ekholm, & Varo, 2000). This variation in MW, together with the structural modifications resulting from the domestic and commercial processing of oats, has a direct impact on some of the properties of the β-glucan (Beer, Wood, Weisz, & Fillion, 1997; Tosh et al., 2010). For instance, manipulating the MW of β-glucan and the particle size of oat particles led to materials with different solubility and viscosity (Wang & Ellis, 2014).

Oat β-glucan has similar solution properties to other types of soluble dietary fibre, such as guar galactomannan, which exist in solution as fluctuating ‘random coils’ of glycan chains (Ellis, Wang, Rayment, Ren, & Ross-Murphy, 2001; Morris, 1992). The solution properties of these conformationally-disordered polysaccharides, specifically their capacity to generate viscosity, are dependent largely on the number (i.e. concentration) and size (i.e. MW) of the polymer chains that become completely hydrated. Thus, the rate and extent of dissolution as well as the concentration and molecular size of the polysaccharide, are strongly linked to their physiological activity (Judd and Ellis, 2005, Wang and Ellis, 2014). As the polymer concentration increases, individual polymer coils interpenetrate to form an entangled network, resulting in an increase in viscosity (Ellis et al., 2001).

The nutritional value and health benefits of oat are now well established, particularly in relation to its β-glucan content and positive effects on lipid metabolism and potential risk-reduction of cardiometabolic diseases (Welch, 2011, Wolever et al., 2010, Wood, 2007). Moreover, it has been previously reported that oat β-glucan attenuates blood cholesterol and lipid concentrations due notably to its capacity of generating highly viscous solutions in the proximal gut (Othman, Moghadasian, & Jones, 2011; Wolever et al., 2010). As explained above, this property relies on the MW and concentration of the β-glucan present in solution. However, the details of the mode of action of this polysaccharide and its behaviour in the gastrointestinal tract are not fully understood. In particular, it is still unknown how much and how quickly β-glucan is released from the cell walls of the oat tissue matrix. Depending on the method used, oat β-glucan can be difficult to extract and the quantity of polymer that solubilises relies on various parameters such as pre-treatment, particle size and the temperature of extraction (Wood, Siddiqui, & Paton, 1978; Zhang, Liang, Pei, Gao, & Zhang, 2009).

The purpose of the current study was to investigate the effects of differences in structure and particle size of selected oat flours and flakes on the dissolution kinetics and solution rheology of β-glucan during aqueous incubation. We have characterised these complex oat materials by analysing their chemical composition, MW of β-glucan and rheological properties. In addition, we have determined, using a new assay, the temporal release of β-glucan (i.e. dissolved polymer) from raw, milled and hydrothermally processed oat materials. The flow behaviour of the released β-glucan was compared with purer forms of the polymer and also guar gum, an endospermic flour of a leguminous seed containing galactomannan, that has been well-characterised. Finally, macro- and microscopic examination of the material before and after incubation was performed to provide some additional insight of the physical changes in the oat materials.

## 2 Materials and methods

### 2.1 Materials

**Fig. 1:**
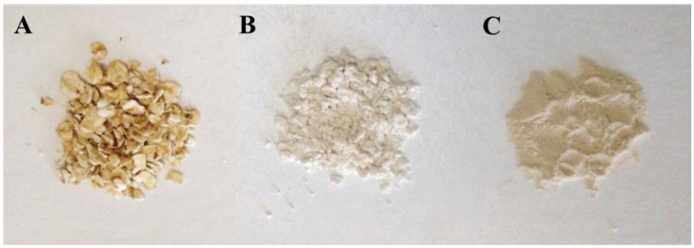
Oat materials used in the study: flakes (A), flour (B) and BG32 (C). Oat flakes are in the mm size range. Average particle size of flour: 60 μm and oat bran BG32: 152 μm.

Oat flakes from the Belinda variety were obtained from Lantmännen Cerealia, Moss, Norway. Oat flour was produced at Nofima from the Belinda oats by milling the flakes on a laboratory hammer mill (Retsch, Model ZM100, Retsch GmbH, Haan, Germany) with a 0.5 mm mesh (Fig. 1). Extracted oat β-glucan of high MW (BG1) was a generous gift from Dr Susan Tosh at Agricultural and Agri-Food Canada. Swedish Oat Fiber (Swedish Oat Fiber AB, Bua, Sweden) provided oat bran rich in β-glucan (BG32) and medium MW β-glucan (BG2). Commercial, food grade guar gum flour (Meyprogat, M150) was generously provided by Dr Graham Sworn (Danisco, Paris, France). Lichenase (EC 3.2.1.73) was purchased from Megazyme (Bray, Wicklow, Ireland) and thermostable *Bacillus licheniformis* α-amylase (Thermamyl^®^ 120) was obtained from Sigma-Aldrich Chemical Co. (Poole, UK). Phosphate buffer (20 mM, pH 6.5) was prepared by dissolving NaH_2_PO_4_ and NaN_3_ (0.02%) in deionised water followed by adjustment of pH with 0.1 M NaOH.

### 2.2 Physical and chemical characterisation of materials

The average particle size of the flours (Fig. 1) was measured using a Malvern laser diffraction particle sizer 2000 equipped with a dispersant unit (Hydro 2000G) filled with water (Malvern Instruments Ltd.). Each oat material and the guar gum (the positive control) were analysed for protein (Kjeldhal method with protein N factor of 5.7), lipid (Soxhlet; hexane), starch (AOAC 996.11), non-starch polysaccharides (Englyst, Quigley, & Hudson, 1994) and β-glucan (AOAC 995.16) content. Moisture (oven-dried at 102 °C) and ash (combustion at 525 °C) contents were also determined. β-Glucans were extracted from the original material using a method previously described (Rieder, Holtekjølen, Sahlstrøm, & Moldestad, 2012) and MW of the extracted β-glucans was analysed with the calcofluor method as described below. β-Glucanase activity in the oat materials (original and incubated samples) was determined using the Megazyme kit assay employing the use of the substrate azo-barley glucan (Megazyme, Product Code: K-MBGL). Duplicate measurements were made for each analysis.

### 2.3 Quantification of β-glucan release

Raw oat material (flakes, flour or BG32) was added to 12 mL of phosphate buffer to obtain a β-glucan concentration of either 0.5 or 1.0% (w/v). Hydrothermally processed (cooked) oat samples of 0.5 or 1.0% (w/v) β-glucan were obtained by adding deionised water (16% of final weight) to the oats, and by placing the samples into a boiling water bath. After 10 min of cooking, 12 mL of phosphate buffer were added to each sample.

The samples were then incubated at 37 °C on a rotator for periods of 0, 5, 10, 15, 30 or 60 min, 2, 5, 8, 24, 48 or 72 h, using one sample per time point. It is still unknown how long the cell walls of plant foods (dietary fibre), such as in oat tissue, remain in the digestive tract before being degraded by bacterial fermentation and, if not totally fermented, excreted in the faeces. Therefore, 72 h was chosen as an end point that represents the maximum time that oats might reside in the gastrointestinal tract. After centrifugation at 1800*g* for 10 min, depending on the viscosity, 0.1–0.5 mL of the supernatant was collected and the β-glucan precipitated in ethanol (two steps: first 95% w/v, then 50% w/v). The extracted β-glucan samples were analysed using an enzymic method based on a cereal mixed-linkage β-glucan kit from Megazyme. The released β-glucan (i.e. solubilised fraction) was expressed as a percentage of total β-glucan originally present in the sample. Each measurement was performed in duplicate. For presentational purposes in the Results section, the experimental data were fitted by non-linear regression using Sigma Plot (version 13 Systat© Software Inc.).

### 2.4 Calcofluor weight-average molecular weight measurements

Calcofluor and cereal β-glucan form a distinct fluorescent complex, which enables the determination of β-glucan MW distributions in the presence of other macromolecules (Wood, 2011, Wood and Fulcher, 1978). In this study, an aqueous size-exclusion chromatographic (SEC) separation of β-glucan with HPLC and an on-line and post-column addition of calcofluor was employed to measure the β-glucan MW in the original oat materials (after extraction as described above) and in the supernatants of the incubated (1, 2, 5 or 72 h) raw or cooked oat samples. Aliquots of the incubated samples were diluted either ten-fold or two-fold in phosphate buffer, depending on β-glucan content. The solution was mixed thoroughly, centrifuged at 1800 g for 10 min, and filtered (0.8 μm syringe filter, Millipore) before injection of 50 μL into the HPLC system as previously described (Rieder et al., 2015b). Briefly, the system consisted of two pumps (UltiMate 3000 pump and degasser module, Thermo Scientific), a Spectaphysics AS3500 auto injector, a guard-column (Tosoh PWXL), two serially connected columns (Tosoh TSK-gel G5000 PWXL and G6000PWXL, maintained at 40 °C) and a fluorescence detector (Shimadzu RF-10A, Shimadzu Europa, Duisburg, Germany). The eluent (50 mM Na_2_SO_4_) was delivered at a flow rate of 0.5 mL/min. Calcofluor (Megazyme) solution (25 mg/L in 0.1 M tris(hydroxymethyl)aminomethane) was delivered post-column through a T-valve at a flow rate of 0.25 mL/min. Fluorescence detection of the formed calcofluor/glucan complexes occurred at λex = 415 nm and λem = 445 nm. A calibration curve for the MW of β-glucan was constructed with in-house β-glucan standards and standards purchased from Megazyme with peak MW from 100 to 1080 × 10^3^ g/mol. A proprietary third order polynomial regression (PSS Poly 3) was fitted to the retention time plotted against the peak MW using PSS WinGPC Unichrome software (PSS, Polymer Standard Service, Mainz, Germany). If not otherwise stated, MW values reported in this study are calcofluor weight-average MW calculated from the measured MW distributions by using PSS WinGPC Unichrome software. Each measurement was performed in duplicate.

It should be noted that outside a MW range of 10–500 × 10^3^ g/mol, the post column calcofluor method yields only relative/apparent molecular weight values (Rieder, Ballance, & Knutsen, 2015). Above a MW of 500 × 10^3^ g/mol, the calcofluor method results in an increasing underestimation of the MW compared to other methods such as SEC-RI, but has the advantage of being able to determine β-glucan MW in crude extracts.

### 2.5 Weight-average molecular weight determination using SEC-MALLS-VISC-RI

The MW distribution of guar galactomannan was determined in aqueous solutions at concentrations of 1 mg/mL as previously described (Rieder et al., 2015a) and is reported as weight-average molecular weight.

### 2.6 Rheological behaviour

Raw and cooked oat samples incubated for 1, 2, 5 and 72 h, as described above (section 2.3.), were centrifuged (1800 g for 10 min) and the supernatant collected for rheological measurements. Also, solutions of purified polymers, guar gum and β-glucan (BG1 and BG2), were used as controls. To ensure total hydration of the polysaccharides, the solutions were prepared by slowly sprinkling the polymer into a rapidly swirling vortex of phosphate buffer and the mixture left to warm up at 80 °C for 2 h, followed by cooling to room temperature overnight. The rheological measurements were carried out on the control and oat samples using a dynamic strain-controlled rheometer (Physica MCR 301, Anton Paar, Stuttgart, Germany) equipped with a double gap geometry (DG 26.7) and a temperature-controlling Peltier unit (C-PTD 200). Viscosity flow curves were obtained in duplicate at 25 °C after 2 min temperature equilibration with the operating shear rate ranging from 0.01 to 1000 s^−1^ with seven measurement points per decade. The measurement point duration ranged from 100 to 1 s during the forward ramp and the backward ramp. The cooked oat samples were recovered after the rheological measurement and treated with thermostable amylase (0.5 mL/g of starch, at 90 °C for 2 h) or lichenase (0.035 mL/mL of β-glucan solution, at 50 °C for 1 h) and the flow behaviour measured a second time. The apparent zero-shear viscosity was estimated by fitting the data to the Cross model (Cross, 1965):

(1) $$\eta =\eta_{\infty }+\left[\eta_{0\text{x}}-\eta_{\infty }\right]/\left[1+{\left(a\dot{\gamma }\right)}^{p}\right]$$

where η_0x_ and η_∞_ are viscosities at zero and infinite shear rate, *a* and $\dot{\gamma }$ are a relaxation time and shear rate, respectively, and *p* is an exponent.

### 2.7 Microstructural characterisation

Raw and cooked particles of oat flour and BG32 were collected at baseline or after 72 h of incubation, mounted and immediately examined with a Leica DMR light microscope (Leica Microsystems Ltd, Lysaker, Norway). Images were captured with a Leica DC3000 CCD camera.

### 2.8 Statistical analysis

The data for β-glucan dissolution were analysed using SPSS version 17.0. For all tests, the significance level was set at *P <* 0.05 (2 tailed) and all data are expressed as means of duplicates. The differences between materials and/or treatments were assessed by one-way analysis of variance (ANOVA) followed by Tukey’s post-hoc test.

## 3 Results and discussion

### 3.1 Characterisation of the studied materials

The purified β-glucan samples contained 87.6 and 82.8% (wet basis) of the polymer for BG1 and BG2, respectively (Table 1). The β-glucan content of oat flakes/flour compared with BG32 was markedly different with samples containing ∼4.5% and 34.8% of β-glucan, respectively. The starch content also differed greatly between the purified β-glucan and the oats, from 60.3% for the oat flakes and flour to 2.4% for BG2. However, the average MW of the β-glucan in BG32 and oat flakes and flour were in a narrow range (∼1080–1120 × 10^3^ g/mol), but much higher than the purified β-glucan samples, BG1 and BG2.

### 3.2 Quantification of β-glucan release

**Fig. 2:**
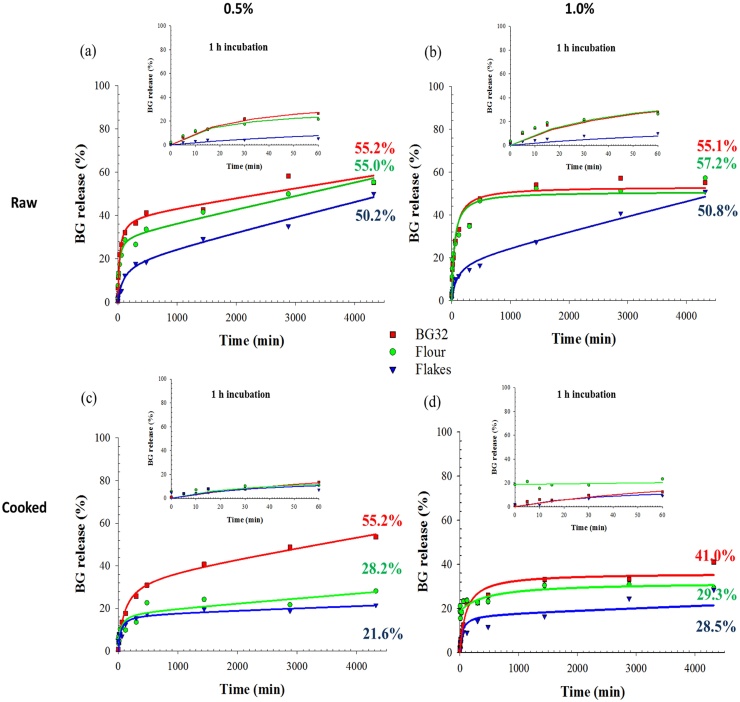
Dissolution kinetic curves of β-glucan released from raw and hydrothermally processed (cooked) oat bran BG32 (red line), oat flour (green line) and oat flakes (blue line) containing either 0.5% (a and c) or 1.0% (b and d) of β-glucan. The data are presented as percentages of the β-glucan originally present in the oat material. The numbers in colour represent the 72 h incubation values, corresponding to the three oat samples. For presentational purposes, the dissolution data were fitted by non-linear regression using Sigmaplot. (For interpretation of the references to colour in this figure legend, the reader is referred to the web version of this article.)

The release of β-glucan from three oat materials, in both raw and cooked states, was investigated by incubating the samples at 37 °C in phosphate buffer for various times, up to 72 h. The rate of β-glucan dissolution varied between the raw oat flakes and the two raw flours (*P <* 0.001) with a more gradual release of the polymer from the flakes (Fig. 2). This can be explained by the differences in particle size (Fig. 1) and therefore surface area to volume ratio. Large particles (mm size range), like the ones from the raw flakes, have a greater proportion of intact cell walls and a longer incubation time is required for the polymer to leach out from the cell walls, especially from walls that are not in close contact with bulk water. Regardless of the oat samples and initial β-glucan concentration, the final values for β-glucan release (72 h incubation) were remarkably close, and were in the range of 50–57%. These results are in agreement with a previous study that showed that after more than two hours of digestion, the solubility of β-glucan from unprocessed oat bran was not complete, and only ∼39% of the polymer was released from the oat tissue (Tosh et al., 2010). In the present study, 2 h of incubation led to a β-glucan release of ∼33, 30 and 12% for BG32, flour and flakes, respectively. The work by Tosh and colleagues also revealed that more processed forms of β-glucan sources showed higher levels of polymer solubility (∼67 to 100%), and the amount released increased with decreasing MW. This observation is compatible with data obtained from a study of hydration kinetics performed on the leguminous cell wall polysaccharide guar galactomannan, which showed an inverse relationship between MW and dissolution rate of the polymer (Wang, Ellis, & Ross-Murphy, 2003). A more recent study also showed incomplete solubility of β-glucan from cereals, including barley, that underwent different forms of processing (Comino, Collins, Lahnstein, & Gidley, 2016).

The hydrothermally treated (cooked) oat flour and flakes (Fig. 2c and d) showed much lower amounts (*P <* 0.001) of β-glucan released after 72 h of incubation (28.8 and 25.1% for flour and flakes, respectively) compared with the raw samples (56.3 and 50.5% for flour and flakes, respectively). The presence of starch could explain this phenomenon, since starch and water-soluble polysaccharides are highly likely to compete for the available free water (Webster, 2011). At the beginning of the hydrothermal process, the starch located on the fractured surfaces of the milled oat particles would have hydrated and swollen by absorbing water. The gelatinisation of the readily available starch on the fractured surface, and in the underlying contiguous cell layers (i.e. the encapsulated starch), are likely to have hindered the release and solubility of the β-glucan. Indeed, it is well known that the physical structure of starches, including oat starch, undergoes rapid and considerable changes when heated at 95 °C, such as swelling and co-leaching of mainly amylose and also some amylopectin (Autio & Eliasson, 2009).

The texture of the oat flakes/flour is also affected by the method of cooking preparation and the resulting starch gelatinisation (Webster, 2011). In general, adding boiling water to the oat flakes will give a grainy texture, while adding cold water, mixing thoroughly and then gradually boiling the flakes (as done in the present study) generates a smoother texture. The preparation method is therefore not without consequences for the release and solubility of β-glucan as revealed by an early study (Yiu, Wood, & Weisz, 1987).

### 3.3 Molecular weight measurements

The results in Table 2 indicate that the MWs of the β-glucan released from the raw and hydrothermally treated oat samples incubated for 1–5 h were similar to the values found in the original non-hydrated oat materials, i.e., ∼1100 × 10^3^ g/mol (see Table 1). Therefore, the β-glucan chains of high MW hydrated relatively rapidly to form polymer solutions in the early stages of incubation without any significant changes in the first 5 h. Prolonged incubation (72 h), however, led to a significant reduction in MW. This is likely to be due to hydrolysis (depolymerisation) of the β-glucan by endogenous β-glucanase as detected by the presence of β-glucanase activity using the Megazyme assay (*data not shown*). The enzyme may have been released therefore at a later stage of incubation because of its likely entrapment within the oat tissue. The cooking method used in the present study did not succeed in fully inhibiting the β-glucanase activity and longer cooking time with stirring may have been more effective, as previously reported (Yiu, 1986, Yiu et al., 1987). Indeed, this method may have permitted starch gelatinisation, but some of the structural integrity of the oat matrix, including the cell walls as well as the native starch structure, appeared to be preserved (see Microscopy results section). The decrease in MW after 72 h, relative to earlier incubation times, was also more noticeable for the flour than the flakes, suggesting perhaps that β-glucanase activity is preserved in the relatively undamaged cells in the inner regions of oat tissue particles, as in the case of the flakes with large particle size. In contrast, the MW of β-glucan in the BG32 sample remained constant throughout the whole incubation time, since this particular flour, enriched in bran, had undergone a processing step used to inactivate β-glucanase.

### 3.4 Rheological behaviour

**Fig. 3:**
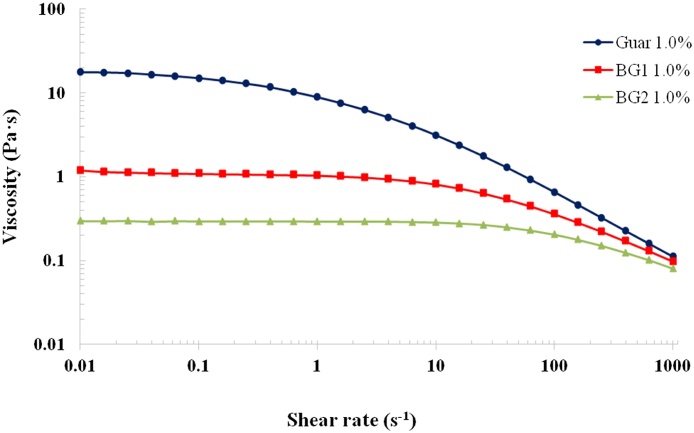
Log-log plot of steady shear viscosity versus shear rate for guar galactomannan and β-glucan (BG1 and BG2) solutions at a concentration of 1.0% (w/v) of polymer.

As previously reported, the fully hydrated polysaccharide solutions (1%, w/v) of guar galactomannan (Rayment, Ross-Murphy, & Ellis, 1995) and the β-glucan samples BG1 and BG2 (Doublier & Wood, 1995; Ren, Ellis, Ross-Murphy, Wang, & Wood, 2003) displayed shear-thinning (pseudoplastic) behaviour with a Newtonian plateau at low shear rates and a shear rate dependence at higher shear rates (Fig. 3). Such solution behaviour is characteristic of semi-flexible polysaccharides and typically described by the entanglement model (Ellis et al., 2001). The guar galactomannan solution showed the highest viscosity values over the range of shear rates measured, followed by lower viscosities (in descending order) for the BG1 and BG2 solutions. These profiles and zero-shear viscosity values (Table S1 of the supplementary material: 18.52, 1.12 and 0.30 Pa·s for guar galactomannan, BG1 and BG2, respectively) are consistent with the MW values reported in Table 1.

**Fig. 4:**
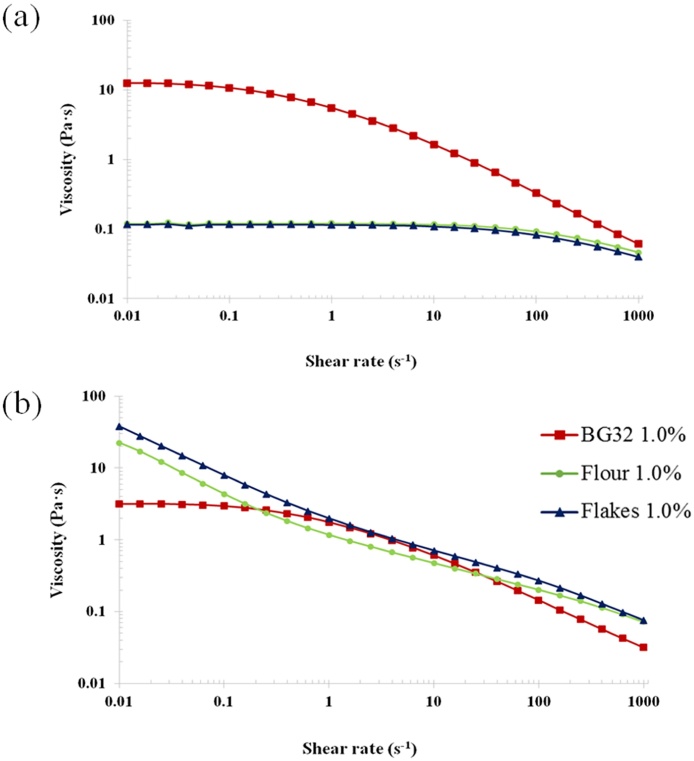
Log-log plot of steady shear viscosity versus shear rate obtained from the β-glucan release experiments (supernatant only) for raw (a) and cooked (b) BG32, oat flakes and oat flour after 72 h of incubation.

The viscosity profiles of the supernatant of the incubated solutions of raw and cooked oat BG32, flakes and flour showed a varied and more complex rheological behaviour than the profiles obtained for the purified polysaccharide solutions (Fig. 4). Thus, despite containing similar amounts of β-glucan at the end of the 72 h incubation period (Fig. 2), large differences were observed in the flow curves of raw BG32, which exhibited the highest viscosities (two orders of magnitude for the Newtonian region), compared with the raw oat flakes and flour (Fig. 4a). Moreover, the zero-shear viscosity values (Supplementary Material; Table S1) show >100-fold difference between these samples. The markedly lower values for the oat flake and flour dispersions after 72 h of incubation are presumably related to the lower MW of the β-glucan contained in these samples, as explained above (Table 2).

The flow curves of the 72 h incubated solutions containing either raw or hydrothermally processed BG32 showed a similar pattern, namely, a Newtonian plateau followed by a shear-thinning region, typical of an entangled polymer solution, although the viscosity values were lower overall after thermal treatment (Fig. 4b). This reduction in viscosity is likely to be due to the smaller proportion of solubilised β-glucan in the 1.0% polymer solution post-cooking compared with the raw samples, as shown by the release experiments (Fig. 2b and d). Factors such as denatured protein and gelatinised starch located on the periphery of the BG32 particles, where the cells are likely to be fractured, may potentially hinder β-glucan release from the cell wall matrix (see Microscopy section below). Furthermore, the cell wall content and structural interactions between the β-glucan and the other cell wall polysaccharides, specifically cellulose and arabinoxylans, are known to vary between different parts of the oat grain, i.e., endosperm versus aleurone layers (Miller and Fulcher, 2011, Wang and Ellis, 2014). Different thermal and mechanical processing conditions are known to affect the properties of oat materials (Yiu, 1986), including the behaviour of the cell wall matrix (Wang & Ellis, 2014). Thus, physical changes during processing are likely to impact on the release and dissolution of the β-glucan during incubation of the BG32, especially if there are alterations in the structure and properties of cell walls that hinder the interaction of β-glucan with the aqueous phase.

**Fig. 5:**
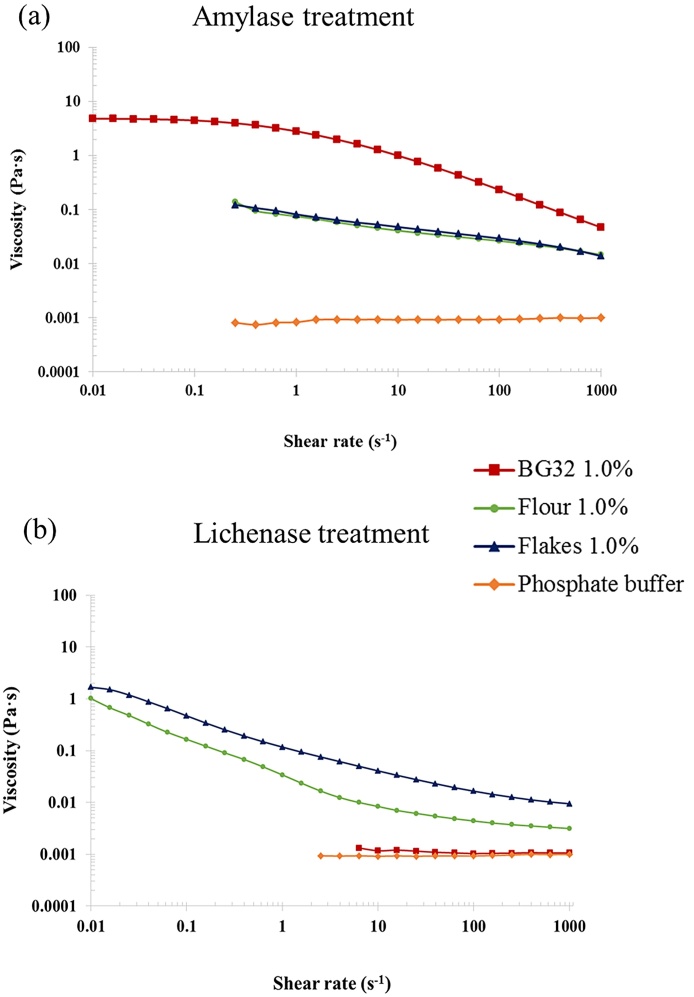
Log-log plot of steady shear viscosity versus shear rate of released β-glucan samples from the cooked oat materials and following treatment with amylase (a) or lichenase (b). The rheological profiles of the untreated samples (controls) are presented in Fig. 4.

As well as significantly inhibiting the release of β-glucan from the oat flakes and flour (Fig. 2), cooking also had marked effects on the rheology of the corresponding samples of incubated solutions. These effects relative to the rheological behaviour of the raw samples, included a substantial increase in viscosity, and the disappearance of the Newtonian plateau at the lowest shear rates of the flakes and flour solutions (Fig. 4). The loss of the Newtonian region and the appearance of a ‘power-law’ behaviour at low shear rates could be attributed to the presence of residual particulates in the samples, in particular starch. This hypothesis is supported by the rheological data for the BG32 sample, which has a substantially lower starch content than the flakes and flour. Thus, solutions of both raw and cooked BG32 displayed similar rheological behaviour typical of an entangled polysaccharide network (Fig. 4). Treatment with amylase or lichenase also allowed us to distinguish between the effects of starch and β-glucan on the solution rheology of oat BG32, flakes and flour by monitoring the changes in viscosity-shear rate profiles (Fig. 5). Amylase addition to the incubated BG32 solution had virtually no effect on viscosity values and shear-thinning behaviour, indicating that the starch in this sample did not contribute to solution rheology (Fig. 5a). Instead, the addition of lichenase (which depolymerises β-glucan) induced a major drop in the viscosity of BG32 (Fig. 5b), which is consistent with the results from a recent study (Gamel, Abdel-Aal, Ames, Duss, & Tosh, 2014). This behaviour provides convincing evidence that the β-glucan content in BG32 is the major contributor to the rheological behaviour of the incubated BG32 solutions. However, in the case of the oat flake and flour solutions, the enzyme treatments induced some decrease in the viscosity compared with the original cooked samples (Fig. 4, Fig. 5). Treatment with amylase had the greatest impact on the rheological properties, with a decrease of several orders of magnitude in the viscosity. This result again confirms the major role of starch in contributing to the rheological behaviour of the incubated oat flakes and flour. Comparison of the solution rheology of flakes and flour (Fig. 4) with the viscosity curves of pure β-glucan (Fig. 3), suggests that the more complex rheological behaviour and higher viscosities of the former samples is attributed mainly to the starch. Other components that may contribute in a minor way to the rheology include proteins and insoluble particulates, such as β-glucan aggregates and micro-fragments of cells and cell walls that remain suspended in the flake/flour solutions.

The effect of these components, including insoluble particulates, on the relative viscosity becomes apparent only when the oat flour and flakes are hydrothermally processed, since starch granules can then swell and gelatinise (Yiu et al., 1987) and proteins can be denatured (Turgeon & Rioux, 2011). The contribution of gelatinised starch to solution rheology in our experiments may have originated from leached polymer (mostly amylose) and/or amylopectin-rich ghosts (Debet & Gidley, 2007). The flake and flour solutions displayed a similar pattern of behaviour to those of model systems of soluble polysaccharides and particulates of different shapes and sizes, namely microcrystalline cellulose and native starch (Rayment et al., 1995; Rayment, Ross-Murphy, & Ellis, 2000). Thus, in a guar galactomannan/rice starch system, with increasing filler (starch) concentrations, an increase in viscosity and greater rate-dependence at low shear rates (i.e. power-law behaviour) were reported (Rayment et al., 1995).

**Fig. 6:**
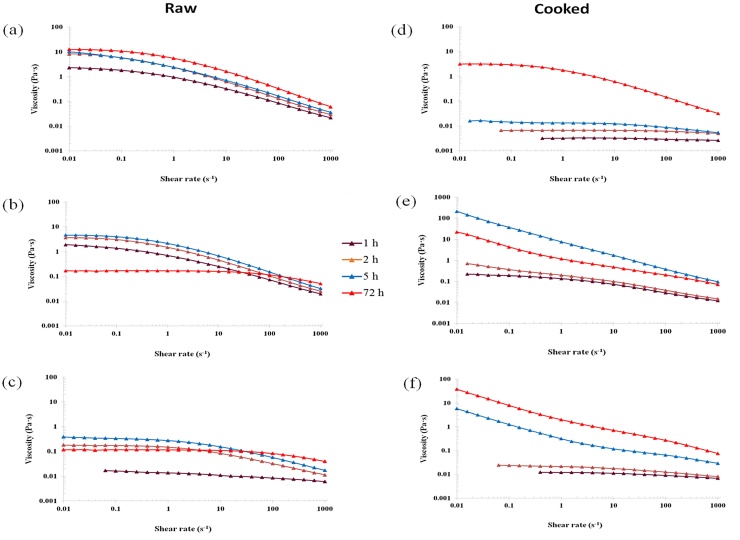
Log-log plot of steady shear viscosity versus shear rate showing flow behaviour of released β-glucan samples from raw BG32 (a), raw flour (b), raw flakes (c), cooked BG32 (d), cooked flour (e) and cooked flakes (f) collected after 1, 2, 5 or 72 h of incubation.

The flow behaviour of the oat solutions incubated for different periods of time (1, 2, 5 or 72 h) are shown in Fig. 6. The trend of the viscosity curves over the range of shear rates measured followed an expected pattern, based on what we observed for the β-glucan release data (Fig. 2), in that viscosity levels increased with increasing incubation time. This relationship is particularly clear-cut for the raw and hydrothermally processed BG32, and for the flakes and flour at early incubation times (1–5 h). However, at the longest incubation times the relationship between viscosity and incubation time is much less clear, as the viscosity curves for some of the 72 h samples were significantly lower than the values obtained at earlier times. As explained in Section 3.3, the retention of β-glucanase activity, which would hydrolyse the β-glucan, occurred in some of the flake and flour samples.

Moreover, the viscosity profiles of the solutions produced from the incubation of raw and cooked oat flakes were markedly lower than the corresponding flour samples milled from the flakes, apart from the 72 h solution (Fig. 6). This suggests that the particle size, and therefore the surface area to volume ratio, of the oat materials had a significant impact on the rate and extent of β-glucan release from the cell walls and the accompanying release of intra-cellular macronutrients (e.g. starch). The kinetics of nutrient release (bioaccessibility) will be highly dependent on the relative proportions of fractured cells in the flour produced by milling, which has important physiological consequences, as recently observed in other plant ingredients and foods (Edwards et al., 2015; Edwards, Warren, Milligan, Butterworth, & Ellis, 2014; Grundy, Wilde, Butterworth, Gray, & Ellis, 2015).

Therefore, as previously demonstrated (Gamel, Abdel-Aal, Wood, Ames, & Tosh, 2012), both the structure of the oat matrix and its composition have an impact on the rheological profile of the ‘solubilised’ material. In the current rheological studies, the properties of the supernatants obtained from incubated and centrifuged oat suspensions were investigated to study the dissolution kinetics of the oat β-glucan (i.e. polymer release into the aqueous phase). Our in vitro data suggest that from a physiological perspective, specifically the flow properties of digesta in the gastrointestinal tract, the contribution of solubilised starch and insoluble particulates (filler) to the solution rheology of dissolved β-glucan from ingested oats, may play an important role. Digesta samples containing high concentrations of starch would be digested in vivo by α-amylase, hence the contribution of starch to the rheology of the system is expected to decrease during transit in the proximal gut. The contribution of other components such as cell walls (dietary fibre), which are not digested, may be significant until the cell wall polysaccharides are fermented in the large intestine.

### 3.5 Macro- and microstructural characteristics of recovered particles (pellet)

Photographs of the sediments of the raw and cooked oats, examined after 72 h incubation and centrifugation to remove the supernatant, are presented in the supplementary material (Fig. S1). The images showed that insoluble particulates could easily be distinguished in all of these samples, and not just in the processed macro-particles of oat flakes; i.e. mm-size range. However, much of the particulate structure in the oat samples appears to have been lost post-cooking. This loss in structure was more noticeable in the oat flour, suggesting that one would expect to see an increased release and solubilisation of components, such as starch and β-glucan. Nevertheless, evidence to show increased β-glucan release was not provided by the hydration experiments (Fig. 2d); indeed, β-glucan release into solution was found to be hindered, probably by the presence of gelatinised starch (see Section 3.2). The images of the flour and flakes also show an increase in volume post-cooking mainly because of water uptake and gelatinisation of the starch, which is a major component of these oat samples (Table 1).

**Fig. 7:**
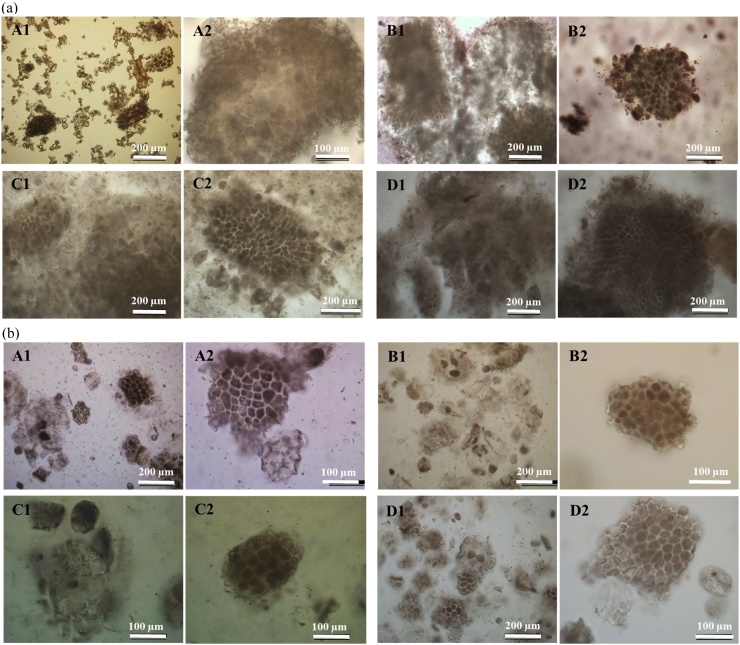
(a) Light microscopy images of raw (A and B) and cooked (C and D) oat flour, at baseline (A and C) and after 72 h of incubation (B and D), and (b) Light microscopy images of raw (A and B) and cooked (C and D) BG32, at baseline (A and C) and after 72 h of incubation (B and D).

Microscopic examination of the two raw flours (oat flour and BG32) revealed the presence of numerous oat tissue particles with seemingly minimal alterations in their structure after 72 h incubation, relative to the pre-incubated baseline (0 h) samples (Fig. 7a and b). Thus, some of these intact oat particles, which varied in size (∼10–200 μm) and contained starch-rich cells of endosperm tissue and β-glucan-rich cell walls, seemed relatively well preserved during incubation, apart from evidence of some swelling and leaching of cell contents. However, the BG32 samples, which are particularly rich in bran, showed incubated particles that appeared even more physically intact than the oat flour, highlighting the robust structure of the tissue. Marked differences in structural characteristics were observed between raw and hydrothermally processed tissues of oat flour. These differences are visible in Fig. 7a (A1 and C1), which show that cooking disrupted the cellular tissue of flour and led to leaching of cellular contents. This leached material formed a swollen network of gelatinised starch together with β-glucan released from the cell walls.

## 4 Conclusions

The novel incubation assay presented in the current work has provided a simple and reproducible method to evaluate the effects of mechanical and hydrothermal processing of oats on oat β-glucan solubility and subsequent solution viscosity. The milling of oat flakes to produce oat flour of smaller particle size increased the rate and extent of release and dissolution of β-glucan from the cell walls of the oat tissue. We have provided evidence that cooking has a significant impact on dissolution kinetics of cell wall β-glucan and its rheological behaviour in solution. Thus, for example, the rate and extent of β-glucan dissolution was severely inhibited by the cooking process in the oat flakes and flour, possibly related to physical hindrance by gelatinised starch. The solutions obtained from cooked flour and flakes displayed complex rheological properties by showing a significant increase in the viscosity profiles, and also a loss of the Newtonian plateau (i.e. power-law behaviour at low shear rates) compared to the raw samples. This behaviour can probably be explained by the contribution of insoluble particulates (e.g. cell fragments and swollen starch granules) and leached amylose. This study also demonstrated that β-glucans from oats, in particular flour, are susceptible to degradation by β-glucanase during incubation, thereby attenuating viscosity, but this occurs only after very prolonged periods of time (72 h).

Therefore, mechanical and hydrothermal processing of oats will strongly influence the release of cell wall β-glucan and intra-cellular nutrients such as starch. This has important implications for the physiological effects of oat β-glucan on gut function, including the rate of gastric emptying, nutrient digestion and absorption, and on subsequent postprandial metabolism (e.g. lipaemia). Variations in β-glucan action, resulting from changes in processing conditions applied to oat products, will significantly impact on a range of related health effects, in particular the cholesterol-lowering property of β-glucan. Our future experiments will focus on investigating the effects of oat structure and β-glucan dissolution properties on the digestion of lipids and other macronutrients under simulated physiological conditions. In addition, the contribution of insoluble particles to the rheological behaviour of β-glucan during simulated digestion warrants investigation to assess their physiological relevance.

## Figures and Tables

**Table 1:** Chemical composition of the guar gum and oat materials^a^, and weight-average molecular weights of the extracted and purified polysaccharides, guar galactomannan and oat β-glucan.

	Guar gum	BG1^b^	BG2^b^	BG32	Oat flake, Oat flour
Protein (N x 5.7) (%)	4.4	–	–	18.9	11.5
Crude lipid (%)	1.0	–	–	3.5	9.6
Starch (%)	0.8	4.2	2.2	7.5	60.3
Non-starch polysaccharides (%)	81.2	76.9	74.6	47.6	6.8
Cellulose (%)	–	0.8	0.7	2.4	0.6
Arabinoxylan (%)	–	6.1	1.5	12.8	1.8
Beta-glucan (%)	–	82.8	87.6	34.8	4.5
Galactomannan (%)	78.2	–	–	–	–
Moisture (%)	11.3	9.8	14.3	8.3	10.4
Ash (%)	0.6	1.7	2.8	3.0	1.7
Weight-average molecular weight (x 10^3^ g/mol)	2500^c^	730^d^	470^d^	1080^d^	1120^d^

−: Not present or in trace amounts only.

a The data are expressed on a wet weight basis. Values are presented as means of duplicates.

b BG1: high MW β-glucan; BG2: medium MW β-glucan.

c Weight-average molecular weight values were determined by SEC-MALLS-VISC-RI.

d Weight-average molecular weight values were determined by the calcofluor method.

**Table 2:** Calcofluor weight-average molecular weights of the β-glucan released after incubation of raw and hydrothermally-processed (cooked) oat materials for 1, 2, 5 and 72 h.

	Weight-average molecular weight (x 10^3^ g/mol)^a^			
	*Raw*		*Cooked*	
	*0.50%*	*1.00%*	*0.50%*	*1.00%*
BG32				
*1* *h*	955	1021	1113	955
*2* *h*	918	1020	1024	1000
*5* *h*	1063	1060	1082	1056
*72* *h*	1093	1048	1155	1136
Flour				
*1* *h*	1113	1076	1039	1073
*2* *h*	1034	1177	1083	1111
*5* *h*	1076	1098	1082	1055
*72* *h*	564	414	568	609
Flakes				
*1* *h*	991	1074	1018	1050
*2* *h*	1000	1120	1098	1103
*5* *h*	1003	1099	1102	1096
*72* *h*	673	488	772	851

a Values are presented as means of duplicates.
