# Supplementary material for: Impact of hydrothermal and mechanical processing on dissolution kinetics and rheology of oat β-glucan
Source: Carbohydr Polym. 2017 Jun 15;166:387–97. doi: 10.1016/j.carbpol.2017.02.077 (PMC5388193; doi:10.1016/j.carbpol.2017.02.077)
Supplement: Supplementary file 1 [file mmc1.docx]

**Appendix A. Supplementary Data**

**Table S1**

Apparent zero-shear viscosities for the extracted and purified polysaccharides, guar galactomannan and oat materials solutions obtained from a fit of the data to the Cross model (see Eq. 1 in main manuscript).

| Sample | Apparent zero-shear viscosity  (Pa·s) | |
| --- | --- | --- |
| Guar gum | 18.52 | |
| BG1 | 1.12 | |
| BG2 | 0.30 | |
|  | *Raw* | *Cooked* |
| BG32 |  |  |
| *1 h* | 2.53 | N/A |
| *2 h* | 9.42 | N/A |
| *5 h* | 14.11 | 0.24 |
| *72 h* | 9.20 | 3.32 |
| Flour |  |  |
| *1 h* | 1.93 | 0.23 |
| *2 h* | 3.94 | N/A |
| *5 h* | 4.90 | N/A |
| *72 h* | 0.08 | N/A |
| Flakes |  |  |
| *1 h* | N/A | 0.01 |
| *2 h* | 0.18 | 0.03 |
| *5 h* | 0.37 | N/A |
| *72 h* | 0.08 | N/A |

N/A: not applicable, since no reasonable fit to the Cross model


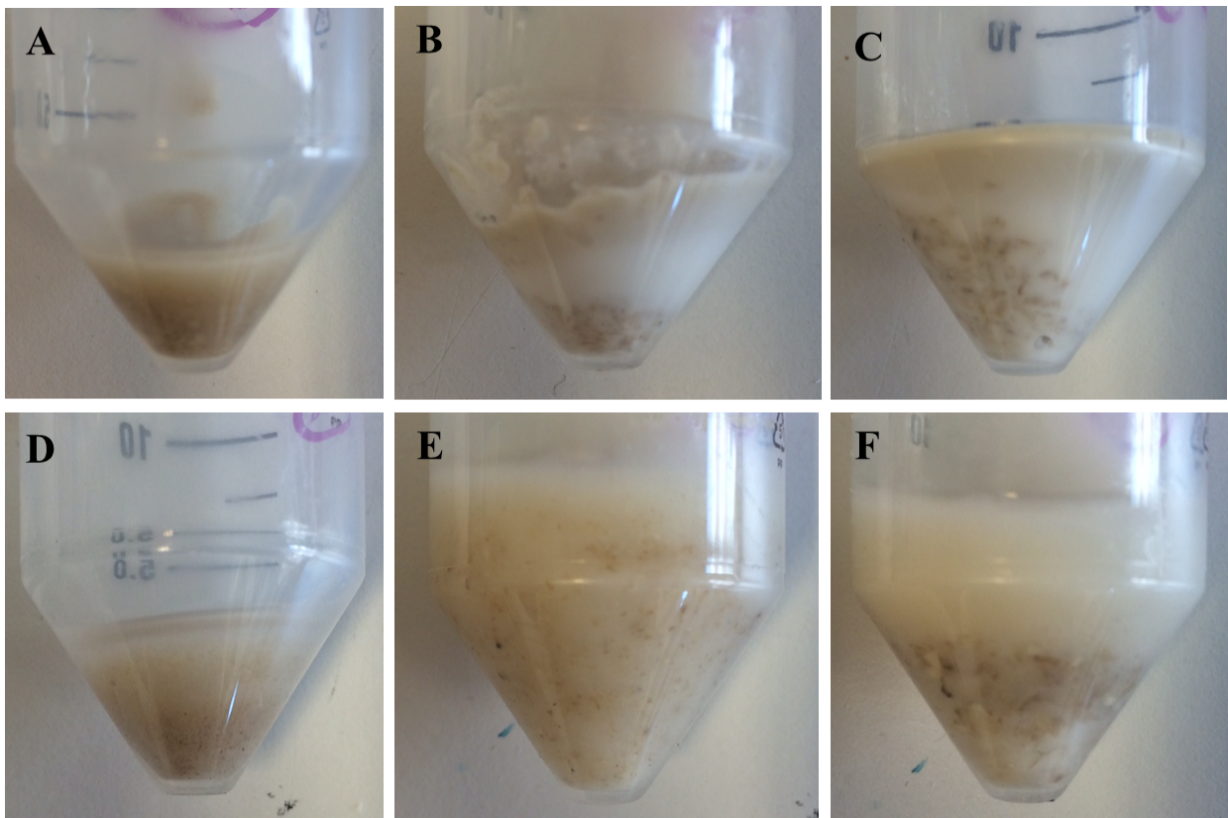


**Fig. S1.** Images of oat materials in raw state (A) BG32, (B) flour, (C) flakes and after hydrothermal processing (cooking) (D) BG32, (E) flour, (F) flakes following 72 h of incubation and centrifugation to remove the supernatant. Images E and F, compared with the corresponding raw samples B and C, respectively, showed an increase in volume due to starch swelling and gelatinisation of flakes and flour (post-cooking). The thick gel/viscous consistency generated post-cooking and seen in images E and F are produced mainly by the gelatinisation of starch, which represents ~60% of the original oat flakes and flour.
